# Avidity characterization of genetically engineered T-cells with novel and established approaches

**DOI:** 10.1186/s12865-016-0162-z

**Published:** 2016-07-13

**Authors:** Victoria Hillerdal, Vanessa F. Boura, Hanna Björkelund, Karl Andersson, Magnus Essand

**Affiliations:** Department of Immunology, Genetics and Pathology, Uppsala University, Uppsala, Sweden; Ridgeview Instruments AB, Vänge, Sweden; Department of Immunology, Genetics and Pathology, Science for Life Laboratory, Uppsala University, SE-75185 Uppsala, Sweden

**Keywords:** T-cell receptor, Affinity, Avidity, LigandTracer, xCELLigence, HLA binding, TARP, CMV pp65

## Abstract

**Background:**

Adoptive transfer of genetically engineered autologous T-cells is becoming a successful therapy for cancer. The avidity of the engineered T-cells is of crucial importance for therapy success. We have in the past cloned a T-cell receptor (TCR) that recognizes an HLA-A2 (MHC class I)-restricted peptide from the prostate and breast cancer- associated antigen TARP. Herein we perform a side-by-side comparison of the TARP-specific TCR (TARP-TCR) with a newly cloned TCR specific for an HLA-A2-restricted peptide from the cytomegalovirus (CMV) pp65 antigen.

**Results:**

Both CD8^+^ T-cells and CD4^+^ T-cells transduced with the HLA-A2-restricted TARP-TCR could readily be detected by multimer analysis, indicating that the binding is rather strong, since binding occured also without the CD8 co-receptor of HLA-A2. Not surprisingly, the TARP-TCR, which is directed against a self-antigen, had weaker binding to the HLA-A2/peptide complex than the CMV pp65-specific TCR (pp65-TCR), which is directed against a viral epitope. Higher peptide concentrations were needed to achieve efficient cytokine release and killing of target cells when the TARP-TCR was used. We further introduce the LigandTracer technology to study cell-cell interactions in real time by evaluating the interaction between TCR-engineered T-cells and peptide-pulsed cancer cells. We were able to successfully detect TCR-engineered T-cell binding kinetics to the target cells. We also used the xCELLigence technology to analyzed cell growth of target cells to assess the killing potency of the TCR-engineered T-cells. T-cells transduced with the pp65 - TCR exhibited more pronounced cytotoxicity, being able to kill their targets at both lower effector to target ratios and lower peptide concentrations.

**Conclusion:**

The combination of binding assay with functional assays yields data suggesting that TARP-TCR-engineered T-cells bind to their target, but need more antigen stimulation compared to the pp65-TCR to achieve full effector response. Nonetheless, we believe that the TARP-TCR is an attractive candidate for immunotherapy development for prostate and/or breast cancer.

## Background

Cancer immunotherapy is becoming a cornerstone in the clinical care of cancer patients due to the breakthrough of immune checkpoint blockade antibodies [1]. Adoptive transfer of ex vivo-engineered autologous T-cells has also been successful, especially when T-cells are engineered with a chimeric antigen receptor (CAR) against CD19 and used for treatment of B cell malignancies [2–5]. A CAR molecule has an extracellular single chain antibody fragment for tumor antigen recognition and intracellular signaling domains from the T-cell receptor (TCR) CD3z chain and co-stimulatory molecules. CAR T-cells provide the advantage of major histocompatibility complex (MHC)-independent binding to tumor-associated antigens on the surface of tumor cells. However, the requirement of surface expression of the antigen limits the available targets making it difficult to identify new and specific CAR targets. Additionally, CAR T-cells do not require very high antigen expression to execute their effector function [6–8], which may be a drawback when targeting antigens that are over-expressed but not restricted to tumor cells.

T-cells engineered with a novel TCR can target antigenic peptides presented by MHC molecules on the surface of tumor cells. This means that also intracellular mutated neoepitopes can be recognized and targeted. As the T-cell naturally uses the TCR for binding, the affinity of the interaction of the introduced TCR would be in the physiological range in which the TCR normally binds. That would at least partly avoid toxicity to normal cells with low expression level of the targeted antigen. As tumor-associated antigens are processed a variety of tumor-associated peptides is available for targeting with TCR-engineered T-cells. TCR-engineered T-cells are now starting to show progress for solid tumors [2]. With novel techniques involving exon and RNA sequencing for identification of mutated neoepitopes, TCR T-cell therapy can be developed on an individual basis [9].

The apparent strength of the interaction between the TCR and MHC/peptide complex is dependent on the affinity, avidity and functional avidity of the TCR for its target. Measuring the dissociation rate or the half-life of a single TCR-MHC/peptide interaction reflects the residence time of the TCR/MHC interaction. Such biochemical measurement is technically challenging and often does not reflect the kinetics of multiple receptor/ligand interactions (avidity), which are needed for the activation of the T-cell. Higher avidity T-cells are found to be more rapidly activated and exert a better cytotoxic function [10], though that is still debated, reviewed in [11]. However, high avidity T-cells may have a lower sensitivity to their ligand. Treating cancer patients with adoptive T-cell therapy that result in targeting normal tissue and severe side effects. In addition, over-stimulated T-cells are short-lived and prone to apoptosis upon antigen engagement. Because TCR-MHC/peptide binding is strengthened by co-receptor binding to MHC, co-receptor expression may affect the subsequent activation of the T-cells. To measure how well T-cells respond to antigen-specific stimulation, the term functional avidity is used. Functional avidity is directly related to the ability of T-cells to adhere to and kill cells expressing the target antigen.

Many reports indicate that virus-specific T-cells are of high affinity (often in the nM range), in contrast to T-cells directed towards the body’s own proteins that are of low affinity due to deletion of high affinity clones in the thymus to avoid targeting self-tissue and autoimmunity [12]. We have cloned two TCRs, one TCR recognizes an HLA-A2-restricted peptides from the prostate and breast cancer-associated antigen TARP [13] and in this paper one TCR recognizing an HLA-A2-restricted peptide from the cytomegalovirus (CMV) pp65 antigen. These TCRs are compared side-by-side. As TARP is an antigen highly expressed in prostate cancer cells it has a potential to be used in the clinics. Therefore, characterization of its affinity, avidity and function as compared to a virus-directed TCR would provide basic information for its effectiveness and safety.

## Methods

### Cells and cell lines

*Target cells*: Mel526 cells (HLA-A2^+^) was cultured in Dulbecco’s Modified Eagle Medium (DMEM), supplemented with 10 % fetal bovine serum (FBS), 1 % penicillin/streptomycin (PEST) and 1 mM sodium pyruvate. T2 cells (HLA-A2^+^) were grown in RPMI-1640 containing 10 % FBS and 1 % PEST. *Producer cells*: HEK-293 T-cells was cultured in DMEM, 10 % FBS, 1 % PEST and 500 μg/ml geneticin (used only during culturing but not during virus production). Peripheral blood mononuclear cells (PBMCs) from buffy coat blood of healthy volunteers (unidentified; bought from the Uppsala University Hospital blood center) were isolated using Ficoll Paque (GE Healthcare, Uppsala, Sweden). PBMCs were cultured in RPMI-1640 supplemented with 10 % human AB-serum (own production), 100 IU/ml interleukin (IL)-2 (Proleukin, Novartis, Basel, Switzerland), 2 mM L-glutamine, and 10 mM HEPES. The cell culture reagents were purchased from Life Technologies (Carlsbad, CA).

### Peptides, peptide pulsing and multimers

The HLA-A2-restricted peptides: TARP(P5L)_4–13_ (amino acid sequence FLPSPLFFFL) [14], CMV pp65_495–503_ (NLVPMVATV) [15] and as negative control VMAT-1_31–39_ (LLLDNMLFT) were synthesized to purity above 95 % (Genscript, Piscataway Township, NJ). Target cells were pulsed for 2 h with peptide at concentrations of 50 μM, 10 μM, 1 μM, 100 nM, 10 nM, 1 nM, 100 pM and 10 pM (only the highest concentration was used for the VMAT-1_31–39_ control peptides). Phycoerythrin (PE)-conjugated TARP(P5L)_4–13_/HLA-A*0201 dextramer was purchased from Immudex (Copenhagen, Denmark) and PE-conjugated pp65_495–503_/HLA-A*0201 tetramer was purchased from MBL International (Woburn, MA).

### TCR cloning and viral vector construction

We have previously reported cloning of the TCR recognizing the TARP_4–13_/HLA-A2 complex. [13] The TCR recognizing the CMV pp65_495–503_/HLA-A2 complex was cloned using the same approach. In brief, PBMCs, isolated from a HLA-A2 positive, CMV-seropositive donor, were stained with the pp65_495–503_ tetramer and tetramer-positive T-cells were isolated using magnetic beads. T-cells were cloned at 0.6 cells/well in 96-well plates and expanded with IL-2 and irradiated feeder cells before testing for peptide reactivity. The TCR chains of ten reactive clones were sequenced and found to contain one unique TCR-α chain and one unique TCR-β chain. A recombinant sequence with the identified TCR α chain linked to the β chain was cloned into a lentiviral vector under the *Spleen Focus-Forming Virus* (SFFV) promoter. The α and β chains were separated by a 2A self-cleaving peptide sequence from *Thosea Asigna Virus* (T2A). Mouse constant domains of TCR α and β were used to improve the pairing between the chains of the introduced TCR chains and avoid mispairing with endogenous TCR α and β chains. Vesicular stomatitis virus (VSV)-G pseudotyped lentiviral particles were produced in HEK 293-T-cells and concentrated by ultracentrifugation as described previously [13].

### T-cell activation, transduction and sorting of TCR-transduced T-cells

T-cells in a pool of freshly isolated PBMCs were activated for 48 h using 100 ng/ml OKT3 antibody (Nordic Biosite, Täby, Sweden) and 100 IU/ml IL-2. One million activated PBMCs were then transduced for 4 h with 50 μl concentrated lentivirus, encoding the pp65-TCR or TARP-TCR as described previously [13]. After transduction the cells were plated in 24-well plates, rested overnight and re-transduced 24 h later. The transduced cells were tested for transduction efficiency using multimers and flow cytometry analysis 7 days after transduction.

To purify TCR-engineered T-cells, the transduced cells were stained with PE-conjugated pp65_495–503_/HLA-A*0201 tetramer or PE-conjugated TARP(P5L)_4–13_/HLA-A*0201 dextramer for 30 min at 4 °C. Anti-PE magnetic beads (Miltenyi Biotec, Bergisch Gladbach, Germany) were then used to separate the PE-labeled T-cells according to manufacturer’s instructions. The purity was estimated by flow cytometry (FACSCanto II BD Biosciences, Franklin Lakes, NJ) using PE-conjugated tetramer/dextramer and antibodies (Biolegend, San Diego, CA) against the following markers: CD3 conjugated with allophycocyanin (APC) or Pacific Blue, CD8 conjugated with fluorescein isothiocyanate (FITC), CD4 conjugated with APC. The results were analyzed using FACS Diva 8 and Flow Jo software (Ashland, OR). The sorted TCR-engineered T-cells were then expanded using a rapid expansion protocol as described earlier [13]. The expanded T-cells then reassessed by flow cytometry and were in all cases found to be > 90 % multimer positive.

### Ligand Tracer® measurement of T-cell binding to target cells

One million mel526 target cells in 2 ml of culture medium were let to adhere overnight to a tilted 10-cm Petri dish. The target cells were then pulsed with peptides as described above. The Petri dish was then inserted on the tilted rotating platform of the Ligand Tracer® instrument (Ridgeway Instruments AB, Uppsala, Sweden) and background measurement of fluorescence was done in real time during rotation (1 rpm) for 30 min.

Transduced and expanded TCR-engineered T-cells were labeled with Carboxyfluorescein succinimidyl ester (CFSE) according to manufacturer’s instructions (Thermo Fisher, Uppsala, Sweden) and then washed thoroughly with serum-containing medium. CFSE-labeled TCR-engineered T-cells (1.5 × 10^5^ cells) were then added to the Petri dish with peptide-pulsed target cells. Rotation started again and T-cell binding (association) to the target cells was measured in real time through detection of fluorescent signal from the target cells (T-cell binding) with subtraction of the fluorescent signal from the opposite side of the Petri dish without target cells. After 90 min another 3 × 10^5^ T-cells were added and the measurement continued.

### ELISA and killing assays

For specific TCR activation experiments, the transduced T-cells were co-cultured with target cells pulsed with relevant peptide and control peptide as described above. To detect IFN-γ secretion, 1 × 10^5^ peptide-pulsed T2 target cells were co-cultured overnight with TCR-engineered T-cells (effector to target ratio 1:1). Supernatants were collected and IFN-γ was measured using ELISA (Mabtech, Nacka Strand, Sweden). For killing assays, 1 × 10^5^ peptide-pulsed luciferase-expressing mel526 target cells were co-cultured with TCR-engineered T-cells at 1:1 ratio (effector to target ratio 1:1) or target cells were pulsed with 10 μM peptide and mixed with effector T-cells at different effector to target ratios (10:1, 5:1, 1:1, 1:5 and 1:10). The results in terms of luciferase activity were analyzed after 72 h using Steady-Glo Luciferase Assay System (Promega, Madison, WI) according to manufacturer’s instructions. For the xCELLigence cell growth assay 2 × 10^4^ mel526 target cells were plated in an E-Plate View (ACEA Biosciences, San Diego, CA). Cell growth was measured overnight using the xCELLigence RCPA DP Instrument (ACEA Biosciences). The next day, the target cells on the E-plate View were pulsed for 2 h with TARP(P5L)_4–13_ or pp65_495–503_ peptide at 10 μM, 100nM or 1nM. The effector T-cells were then added to the target cells (approximately 26 h after plating of the target cells) in 1:1, 5:1 and 10:1 effector to target ratios and the killing of the target cells was continuously measured for an additional 48 h.

## Results

### T-cells can efficiently be engineered to express TCRs recognizing HLA-A2-restricted TARP or CMV pp65 peptides

**Fig. 1:**
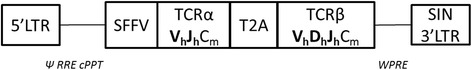
Lentiviral vector for expression of recombinant TCRs. A self-inactivating lentiviral vector was constructed with recombinant TCR α and β chains with human variable (V), diversity (D) and joining (J) gene segments and mouse constant (C) domains. The TCR α and β chains are separated by a self-cleaving T2A peptide and the SFFV promoter controls TCR expression. Lentivirus particles were produced in 293 T-cells and concentrated by ultracentrifugation before being used to transduce human T-cells. Abbreviations: TCR = T-cell receptor; LTR = long terminal repeat; SIN = self inactivating (deletion of the direct repeat enhancer); SSFV = spleen-focus forming virus; V = variable, D = diversity and J = joining gene segment; T2A = 2A peptide derived from *Thosea asigna* virus

The TCR against the HLA-A2/pp65_495–503_ complex was cloned into a lentiviral vector and compared with a lentiviral vector having the TCR against the HLA-A2/TARP_4–13_ complex (Fig. 1). Peripheral blood mononuclear cells (PBMCs) were isolated from buffy coat blood of healthy donor. T-cells in the PBMC pool were activated and then transduced with the TCR-encoding lentiviral vectors. The TCR-engineered T-cells then served as a model system to understand the complex interactions between MHC/peptide and TCR and to compare their ability to bind and respond to target cells.

**Fig. 2:**
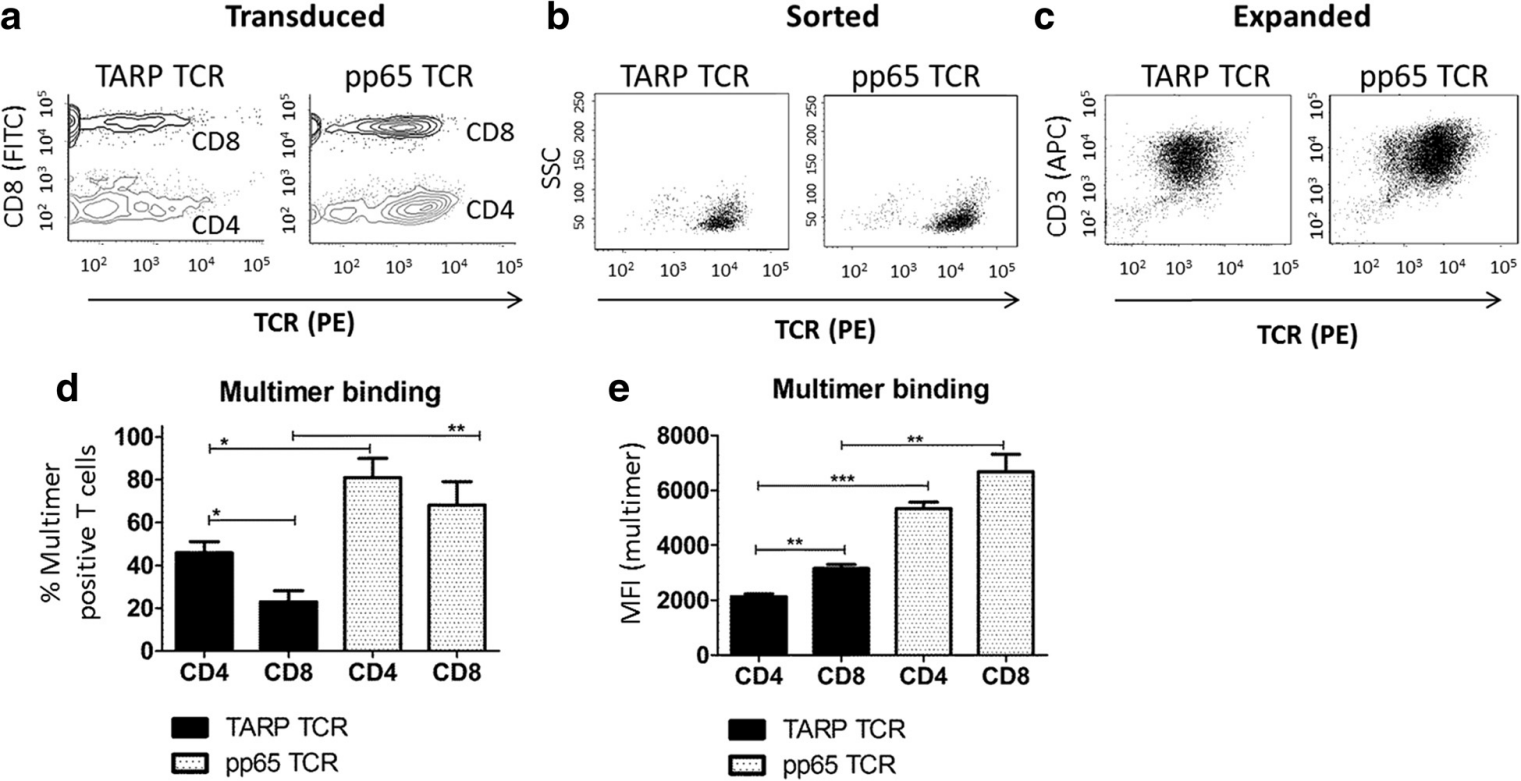
Analysis of TCR-engineered T-cells. Human T-cells in a PBMC pool from healthy volunteers were activated and transduced with the pp65-TCR and TARP-TCR, respectively. The CD3^+^ T-cells were then evaluated for TCR expression by flow cytometry using multimer technologies and CD8 and CD4 staining. Expression of the transferred TCR was assessed seven days after lentiviral transduction (**a**), after multimer-guided magnetic bead sorting (**b**) and after an additional fourteen days of rapid expansion (**c**). The T-cells where analyzed after expansion in terms of percent TCR positive T-cells (**d**) and mean fluorescent intensity (**e**). SSC = side scatter

Several groups have used multimer technology to assess the binding strength of a certain TCR against an MHC/peptide complex. [16–18] To determine how well the CMV pp65-specific TCR (pp65-TCR) and TARP-specific TCR (TARP-TCR) bind to their corresponding HLA-A2/peptide complex, T-cells transduced with the TCRs were analyzed and compared using multimers/flow cytometry and the mean fluorescent intensity (MFI) was used as a measure of their binding strength. Examples from representative experiments showing multimer binding seven days after transduction (Fig. 2a), followed by subsequent multimer/PE-bead sorting (Fig. 2b) and after another fourteen days of rapid expansion (Fig. 2c). The average percentage of multimer positive CD4^+^ T-cells and CD8^+^ T-cells prepared from 3 donors is shown in Fig. 2d. More T-cells were transduced with the pp65 TCR than with the TARP-TCR and we found more CD4^+^ T-cells than CD8^+^ T-cells for both TCR transduction. A higher MFI was also observed for pp65-TCR T-cells than for TARP-TCR T-cells (Fig. 2e), indicating stronger binding, both for TCR-engineered CD8^+^ and CD4^+^ T-cells. The binding of the TCR to MHC/peptide complex is influenced by many factors, one of which is the presence of co-receptor (CD8 or CD4) binding to the conserved part of MHC to stabilize the complex. [19–21] The pp65-TCR and TARP-TCR are binding peptides presented by HLA-A2 (MHC class I) for which CD8 is the co-receptor. Higher MFI for CD8^+^ T-cells than CD4^+^ T-cells probably reflects the influence of the CD8 co-receptor for the MHC class I restricted TCRs. High-affinity TCRs are less dependent on co-receptor binding than low-affinity TCRs. Multimer positivity on TCR-engineered CD4^+^ T-cells therefore indicates a rather high TCR affinity also for the TARP-TCR.

### TCR-engineered T-cell binding to target cells can be studied in real time using the Ligand Tracer Technology

**Fig. 3:**
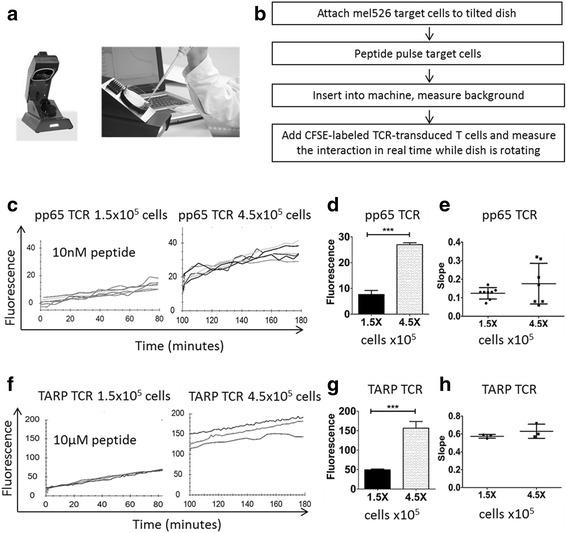
Real time analysis of TCR T-cell binding to peptide-presenting target cells. LigandTracer® (**a**) was used to assess the binding of pp65-TCR and TARP-TCR-engineered human T-cells to HLA-A2 positive target cells (mel526) presenting the cognate peptide (**b**). Target cells were adhered over-night to one side of a Petri dish and then pulsed with either TARP_4–13_ (10 μM] or pp65_495–503_ (10 nM). Sorted and expanded pp65-TCR and TARP-TCR-engineered T-cells were labeled with CFSE and washed before being added to the dish with peptide-pulsed target cells. Binding of TCR-engineered T-cells to peptide-pulsed target cells was measured in real time for 90 min using 1.5 × 10^5^ T-cells and an additional 90 min using 4.5 × 10^5^. Binding kinetics in terms of fluorescence signal from target cells is shown for pp65-TCR T-cells (**c**), with proportional increase of signal when three times more T-cells were added (**d**), and representation of the binding slope (**e**). Binding kinetics in terms of fluorescence signal from target cells is shown for TARP-TCR T-cells (**f**), with proportional increase of signal when three times more T-cells were added (**g**), and representation of the binding slope (**h**)

To understand the kinetics of T-cell binding to target cells, LigandTracer® technology was used (Fig. 3a). A short description of the protocol is outlined in Fig. 3b. Target cells, in our case the HLA-A2^+^ Mel526 cells, are adhered to one side of a tilted Petri dish in an incubator over night and in the morning pulsed with peptide, in our case HLA-A2 restricted TARP_4–13_ or pp65_495–503_. The Petri dish with pulsed and washed cells is then inserted to the round tilted platform of LigandTracer. As the dish rotates, fluorescent detectors at the top of the instrument (where essentially no medium is present because of the tilting) measure the fluorescent signals. When fluorescently CFSE-labeled effector T-cells are added to the medium, a fluorescence signal is detected as they bind to the target cells. Since the target cells are only plated at one side of the rotating Petri dish, TCR binding to MHC/peptide is measured as the difference in fluorescence signal between the signal detected at the target cells and the background signal on the opposite side of the dish (no target cells). The peptide concentrations that were optimal for activation were determined by classical functional assays described below. We pulsed mel526 target cells with 10 μM TARP_4–13_ or 10 nm pp65_495–503_ peptide. At the beginning of the experiment 1.5 × 10^5^ T-cells were added. The initial binding was linear, and the measurement was conducted for 90 min (Fig. 3c, f). At 90 min, 3 times more T-cells (4.5 × 10^5^) were added and the fluorescence continued to increase proportionally to the number of cells (Fig. 3c, f). In both measurements (when pp65-specific or TARP-specific T-cells were used) the fluorescence maximum was three times higher with three times higher cell amount (Fig. 3d, g). There was a bigger variation in the slope when additional cells were added compared to the initial binding curves (Fig. 3e, h). This could be due to the nature of the process of binding, which is accompanied with killing of target cells that would eventually lead to detachment of the effector cells. The rate of detachment would depend on the particular speed of apoptosis induced in the individual cells and this may vary.

### T-cells engineered to recognize the foreign CMV pp65 antigen kill target cells more efficiently than T-cells engineered to recognize the self-antigen TARP

**Fig. 4:**
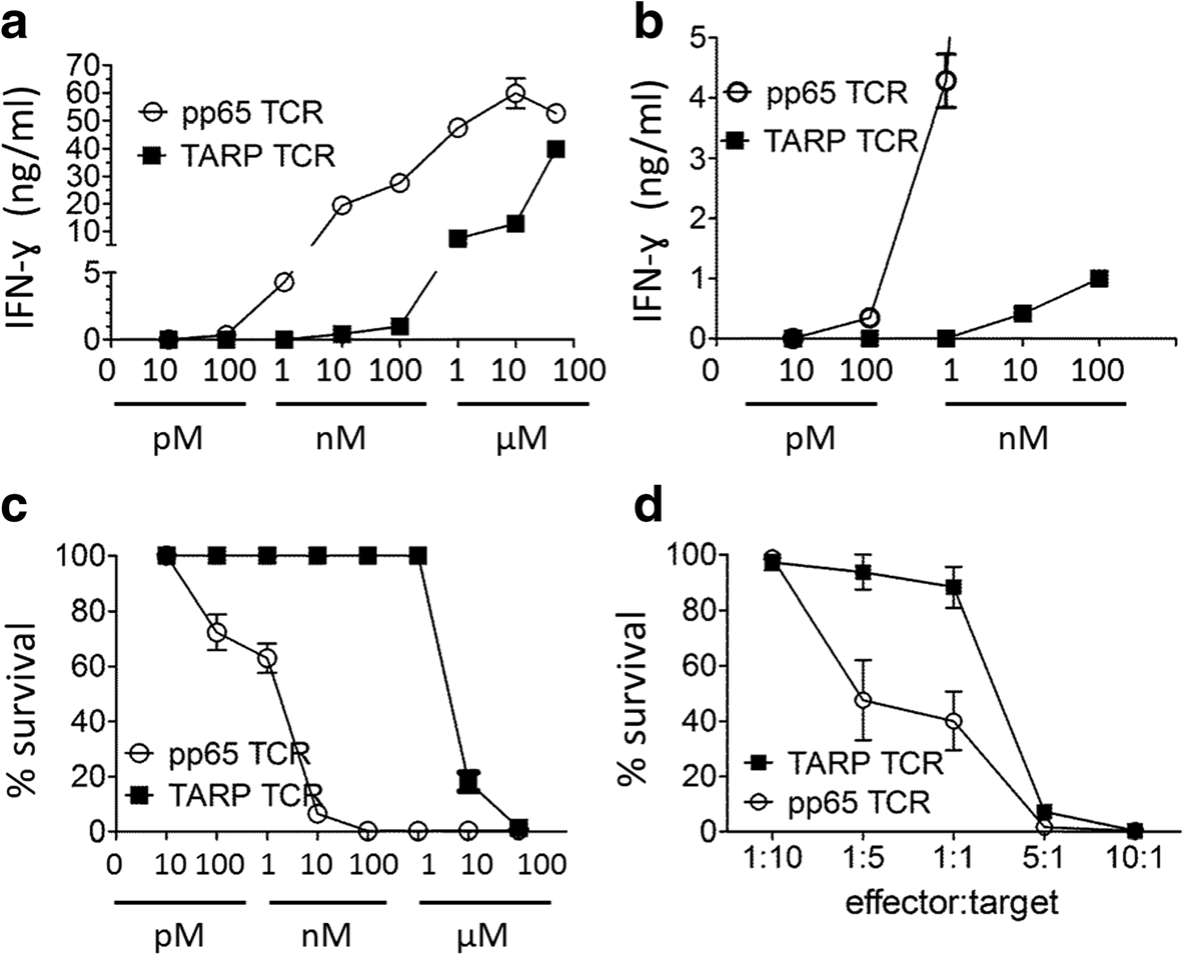
pp65-TCR and TARP-TCR-engineered T-cells show therapeutic effect when encountering HLA-A2 positive target cells presenting the cognate peptide. Sorted and expanded pp65-TCR and TARP-TCR-engineered human T-cells were assayed for response in IFN-γ production (**a**, magnified in **b**) and killing (**c**) of HLA-A2^+^ mel526 target cells pulsed with a range of peptide concentrations. Inactivation of target cells was also assayed at various effector to target cell ratios with target cells pulsed with 10 μM peptide (**d**)

To compare the differences in functional avidity, the performance of the TCR-engineered T-cells was studied in effector functional assays. In the interferon (IFN)-γ assays, the pp65-specific T-cells were superior to the TARP-specific T-cells (Fig. 4a). They required lower peptide amount concentrations (about 1 nanomolar) in order to be activated and produce IFN-γ, in contrast to TARP-specific T-cells, which required a minimum of 100 nM peptide to produce significant amount of IFN-γ (Fig. 4b). Cytokine production is an important effector function, but it does not directly measure the ability of the cytotoxic T-cells to kill target cells. In some cases, primarily in the setting of virus-specific T-cells, IFN-γ producing cells may fail to exert a potent cytotoxic function [22–24]. To further analyze the ability of the TCR-engineered T-cells to respond to antigen stimulation, the pp65-TCR and TARP-TCR T-cells were compared in killing assays using luciferase-tagged target cells. We hypothesized that more functionally effective T-cells would respond to smaller antigen doses, and it also would take less number of effector cells to neutralize the same number of target cells. TARP-specific T-cells killed efficiently their targets at 10 μM peptide (Fig. 4c), and at effector to target ratios of 5:1 when the highest peptide concentration was used (Fig. 4d). In contrast, the pp65-specific T-cells started to exert their cytotoxic function already at 100 pM and efficient killing was achieved at peptide concentration of 10 nM (Fig. 4c). Furthermore, at effector to target ratio of 1:5, approximately half of the target cells underwent apoptosis. Both TCRs were effective at high concentrations of peptide.

**Fig. 5:**
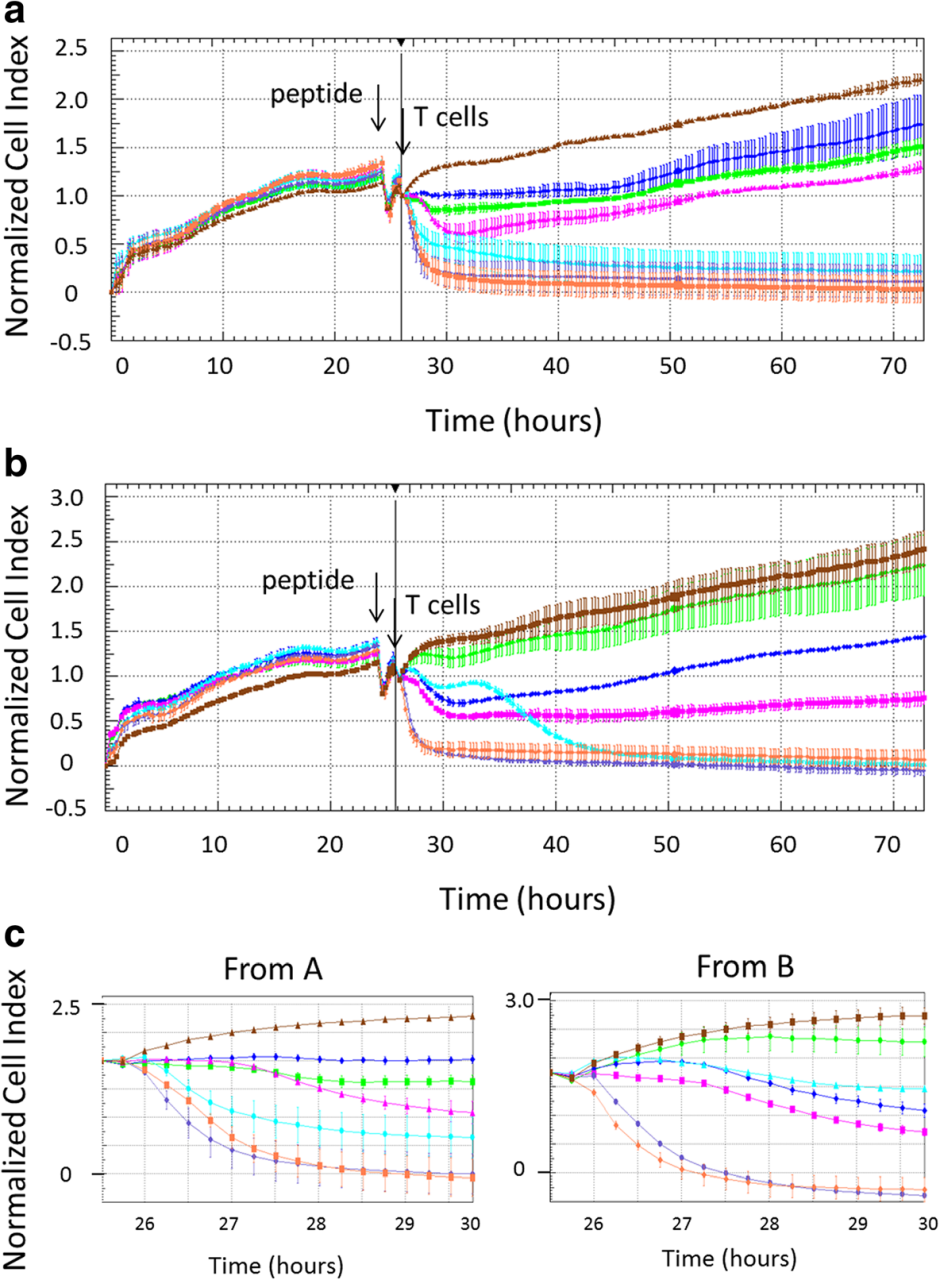
Target cell death in response to pp65-TCR and TARP-TCR-engineered T-cells in real time. Mel526 target cells were plated in E-Plate View and continuously monitored for growth using the xCELLigence DP Instrument for approximately 24 h. **a** The mel526 target cells in the E-Plate View were then pulsed with pp65_495–503_ peptide at 10 μM (Slateblue), 100 nM (Coral) or 1nM (Cyan), or TARP_4–13_ peptide at 10 μM (Magenta), 100 nM (Green) or 1 nM (Blue), or irrelevant VMAT-1_31–39_ peptide as a control (Saddlebrown) for 2 h. The pp65-TCR or TARP-TCR-engineered T-cells were then added at effector to target cell ratio of 5:1. The Cell Index measured was normalized to the time point of T-cell addition. Arrows indicate peptide and T-cell addition. **b** The mel526 target cells in the E-Plate View were pulsed with pp65_495–503_ and TARP_4–13_ peptide at 10 μM for 2 h. pp65-TCR T-cells were then added in effector to target ratios 1:1 (Cyan), 5:1 (Coral) or 10:1 (Slateblue) or TARP-TCR T-cells were added at ratios 1:1 (Green), 5:1 (Blue) or 10:1 (Magenta), or no T-cells were added as a control (Saddlebrown). Arrows indicate peptide and T-cell addition. **c** Growth curves between 26 and 30 h of measurement, i.e., 0 to 4 h post addition of T-cells, are magnified from A (Left) and B (right)

To monitor target cell killing in real time, we used the xCELLigence technology, which measures cell growth and proliferation through electrical impedance measurements. In this assay, impedance was measured every 15 min for a total of 72 h. By measuring the growth of peptide-pulsed mel526 target cells in presence of T-cells, the T-cell cytotoxicity was estimated. The mel526 cell growth was measured for 24 h before TCR-engineered T-cells were added and then measured for an additional 48 h. TARP-TCR T-cells killed their targets less efficient and slower than pp65-TCR T-cells. We observed a response in relation to the amount of peptide added for both pp65-TCR T-cells and TARP-TCR T-cells (Fig. 5a). When target cells were pulsed with 10 μM peptide and effector cells were added at different effector to target ratio, a T-cell dose-dependent response was observed (Fig. 5b). That was more apparent for the TARP-TCR T-cells where there was an improvement of killing with T-cell dose, whereas for pp65-TCR T-cells there was improvement of response when increasing the effector to target cell ratio from 1:1 to 5:1, but at effector to target ratio 5:1 the T-cell response was already saturated (Fig. 5b). A magnification of T-cell killing during the first four hours are shown in Fig. 5c.

## Discussion

We have previously identified a novel TCR against an HLA-A2-restricted peptide from the prostate and breast cancer-associated antigen TARP [13] and in this study, we have further characterized its interaction and binding to HLA-A2^+^ targets cells. We also report the cloning of a TCR against an HLA-A2-restricted peptide from CMV pp65 and demonstrate its strong ability to recognize and kill pp65-pulsed HLA-A2^+^ target cells. The purpose of this study was to investigate the difference in target recognition between T-cells with a virus-directed TCR (pp65-specific) and tumor self antigen-directed TCR (TARP-specific) using both established and novel analyses methods.

It is known that high and low affinity TCRs needs different peptide concentrations to exert cytotoxicity and we predicted that to be the case for the pp65-specific and TARP-specific TCRs. From our functional in vitro studies (IFN-γ release and cytotoxicity) it is clear that T-cells with the pp65-specific TCR respond to a lower peptide concentration than T-cells with the TARP-specific TCR. We verified that pp65-TCR T-cells were also able to kill target cells at low peptide concentration (100 pM) as previously described for T-cells specific to a different antigen [25, 26]. The bioinformatics predictive value of the binding strength of the peptide to HLA-A2, as estimated by NetMHC was comparable for the TARP(P5L)_4–13_ or pp65_495–503_ peptides. Both were categorized as strong binders. Therefore, we believe that a direct comparison between the two TCRs is valid.

When using multimers to assess the TCRs we found that the pp65-TCR resulted in a higher transduction rate (%-wise) of T-cells and that the T-cells had higher MFI than the TARP-TCR. There were slightly more CD4^+^ T-cells than CD8^+^ T-cells that were multimer positive for both TCRs. The reason for this is unknown but it may indicate a slight preference of transducing CD4^+^ T-cells over CD8^+^ T-cells, that CD4^+^ T-cells have better viability after transduction with lentiviral vectors or simply this occurs because there are more CD4^+^ T-cells than CD8^+^ T-cells in peripheral blood. CD8^+^ T-cells had a higher MFI than CD4^+^ T-cells. This is expected as the transferred TCRs interact with HLA-A2 (MHC class I) for which CD8 is the co-receptor. The fact that CD4^+^ T-cells could also be detected by multimers indicates that both TCRs had a rather high affinity since they could be detected without the CD8 co-receptor. In the therapeutic setting of adoptive T-cell transfer it may be advantageous to have T-cells that have a good binding for the target antigen but do not get activated too rapidly, as that may lead to their exhaustion.

When using IFN-γ release and in vitro killing assays pp65-TCR T-cells were superior to TARP-TCR T-cells. This is probably due to higher affinity. TARP is a self-antigen and the TARP-TCR was cloned from a healthy HLA-A2 positive donor [13]. Although TARP has restricted expression in differentiated cells it is possible that the TARP_4–13_ epitope has been presented by thymic antigen-presenting cells during T-cell development and the high affinity T-cell clones against it were deleted during negative selection. Most T-cells recognizing self-antigens (including tumor-associated self antigens) are of lower affinity and avidity or anergic. A recent study, addressing the responses of several TCRs directed against tumor-associated antigens, concludes that T2 cells pulsed with nanomolar concentrations of peptide generally corresponds to the number of epitopes that are physiologically presented by tumor cells [27]. For the TCRs tested in that study, partial response was detected at 10 nM peptide for all antigens tested in terms of ELISPOT IFN-γ production [27]. Although it is difficult to directly compare our results with theirs, small amounts of IFN-γ were detected in our study at 10 nM TARP peptide by ELISA. Of note, the amounts detected exceeded the limit for reactive clones that is used in adoptive T-cell transfer of expanded tumor-infiltrating lymphocytes to cancer patients of some investigators [28]. One also need to keep in mind that T-cells with a high affinity TCR have caused severe side effects in experimental treatment of cancer with adoptively transferred T-cells [29]. One suggested mechanism is through TCR signaling enhancement through Tim-3 [30].

In this study, we introduce LigandTracer technology as a method to monitor the binding of T-cells to target cells in real time. It is a convenient method to demonstrate the increase in the binding as the signal is proportional to the number of the cells and increases in a linear fashion. TCR binding to the cognate peptide was demonstrated both for the TARP-TCR and pp65-TCR but peptide concentration on target cells needs to be adjusted for individual TCRs for optimal result. It is therefore difficult to directly compare the pp65-specific T-cells and the TARP-specific T-cells. However, LigandTracer data suggest that the better in vitro performance of the pp65-TCR T-cells (IFN-γ release and cytotoxicity) cannot be explained solely with better binding to target cells. To complement the binding data from the LigandTracer experiments, we looked at target cell inactivation in real time using the xCELLigence cell growth assay technology to assess the killing potency of the TCR-engineered T-cells. The TARP-TCR T-cells were less efficient in killing their targets and required both higher peptide concentrations and higher effector to target ratios to achieve efficient response. The killing kinetics differed between the TARP-TCR T-cells and pp65-TCR T-cells, and it may indicate that there are different mechanisms that are employed if the T-cell-mediated killing is slower. The TARP-TCR T-cells were able to kill target cells, which growth plateaued at Cell Index of approximately 1, whereas pp65-TCR T-cells had fast and efficient killing shown by the Cell Index going back to 0. For TARP-TCR T-cells, at all peptide concentrations, and for pp65-TCR T-cells, at a low peptide concentration, targets cells started to grow back again, but were then killed in something that looks like a second wave of T-cell killing. In the case of pp65-TCR T-cells, after the re-growth of target cells, T-cells took control again and the Cell Index dropped to 0. Taken together the data indicate a more rapid and efficient response of pp65-TCR T-cells, and that dependent on the TCR the T-cells may need more time to kill their target cells. It is also possible that because of different binding avidity the target cells could undergo apoptosis at a different speed. Because of the real time fine resolution of the xCELLigence assay (Fig. 5c) and possibility to long term follow up (Fig. 5a, b) we suggest that this assay can efficiently replace the standard ^51^Cr-release assay for T-cell mediated killing.

## Conclusions

This paper includes new assays for T-cell binding to target cells and T-cell killing of target cells that can become useful tools to study the kinetics of T-cell mediated killing. The pp65-TCR cloned in this paper has high affinity and T-cells engineered with this TCR can efficiently kill target cells, indicating that the pp65-TCR may be used to produce T-cells to treat CMV reactivation after transplantation in HLA-A2 recipients. Previous data published by us [13] together with data presented in this paper indicate that the TARP-TCR may have an appropriate affinity for TCR-based T-cell immunotherapy. TARP-directed T-cells bound their targets well but had a less potent effector function than pp65-directed T-cells. Further in vivo experiments may elucidate the physiological strength of its effector function and determine whether affinity enhancement of the TCR would be necessary in order to achieve a better cytotoxic response without compromising its specificity.

## Abbreviations

CAR, chimeric antigen receptor; CD, cluster of differentiation; CFSE, carboxyfluorescein succinimidyl ester; CMV, cytomegalovirus; DMEM, Dulbecco’s Modified Eagle Medium; ELISA, enzyme-linked immunosorbent assay; ELISPOT, enzyme-linked immunosorbent spot-forming cell assay; FACS, fluorescence-activated cell sorting; HLA, human leukocyte antigen; IFN, interferon; IL, interleukin; MHC, major histocompatibility complex; PBMC, peripheral blood mononuclear cells; PE, phycoerythrin; TARP, T-cell receptor gamma alternate reading frame protein; TCR, T-cell receptor; VSV-G, Vesicular stomatitis virus glycoprotein
